# Supplementary material for: Evolution of MALDI-TOF MS Profiles from Lice and Fleas Preserved in Alcohol over Time
Source: Insects. 2023 Oct 20;14(10):825. doi: 10.3390/insects14100825 (PMC10607003; doi:10.3390/insects14100825)
Supplement: Supplementary file 1 [file insects-14-00825-s001.zip › insects-2652489-supplementary-1.pdf]

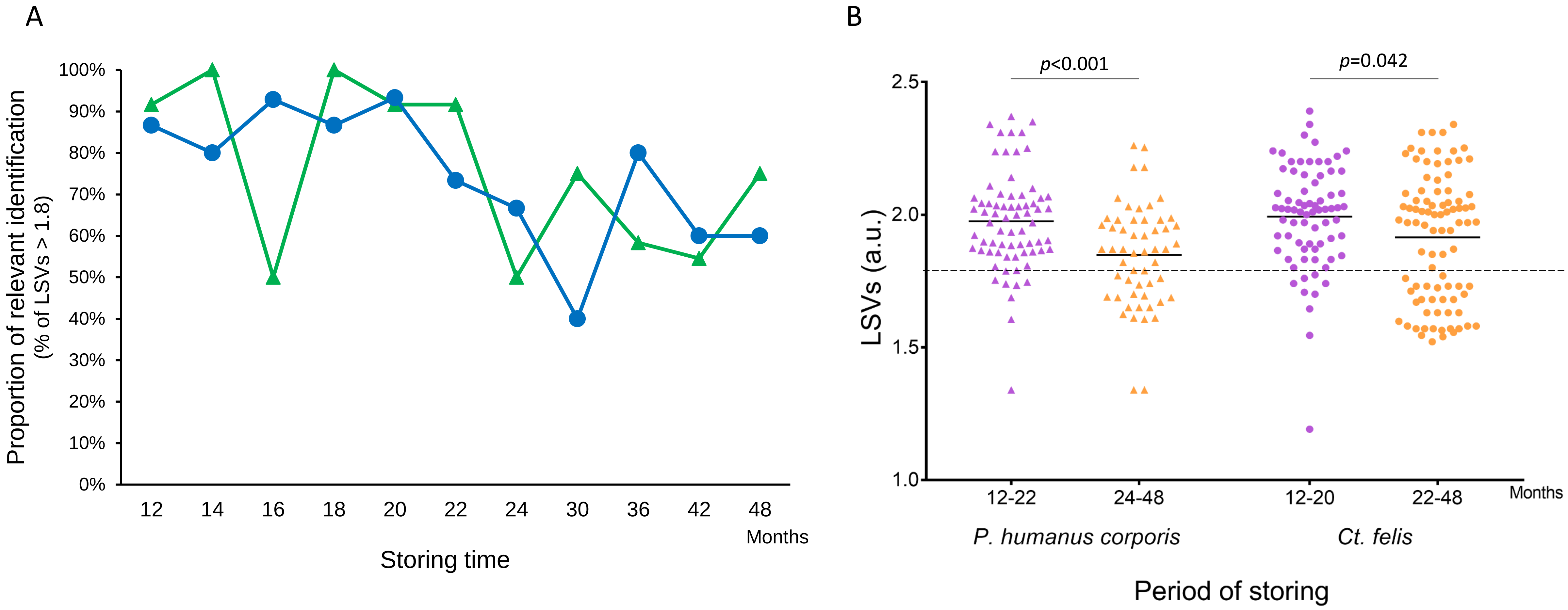

Supplementary File S1. Evolution of the relevant identification of lice and fleas according to the length of storage in alcohol. (**A**) Proportion of *P. humanus corporis* (green line) and *Ct. felis* (blue line) specimens relevantly identified according to the length of storage in alcohol. (**B**) Comparison of the LSVs from *P. humanus corporis* stored in alcohol for 12–22 months and 24–48 months, and *Ct. felis* stored in alcohol for 12–20 months and 22–48 months. Significant differences in the LSVs obtained are indicated (Mann-Whitney test). The dashed line represents the threshold value for reliable identification (LSV > 1.8). a.u., arbitrary units; LSV, log score value.

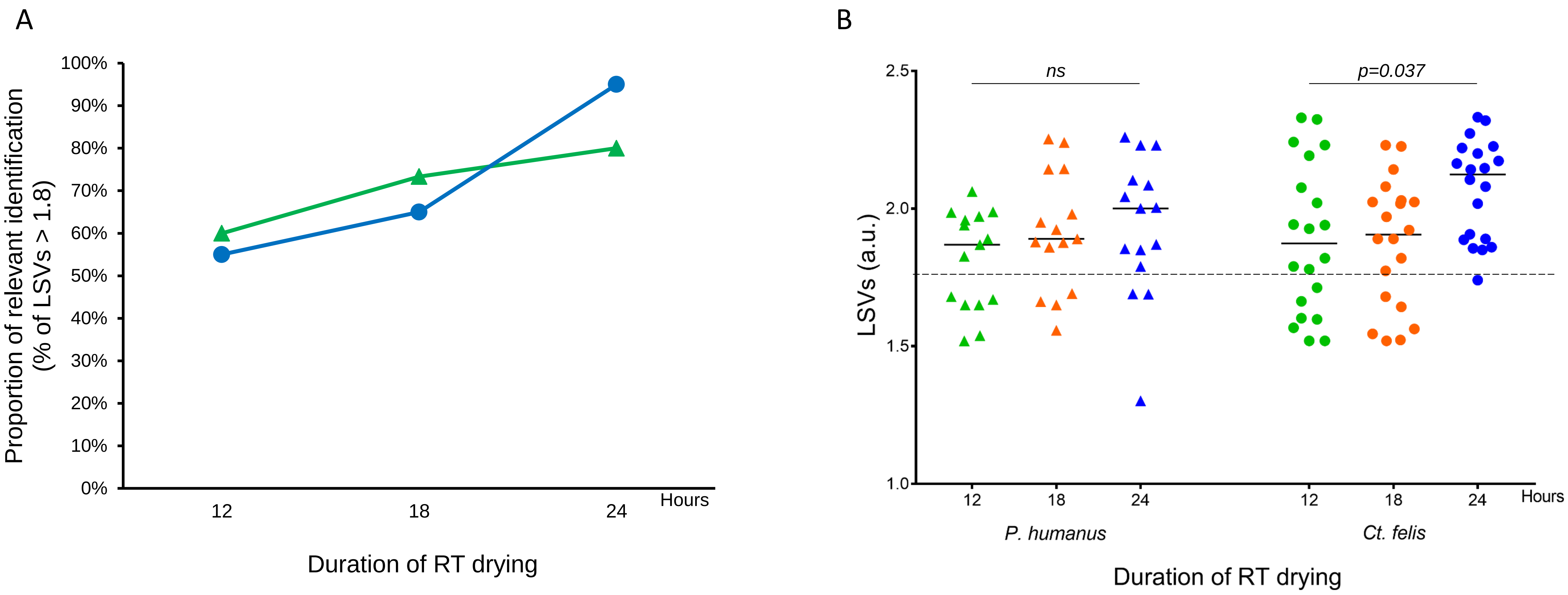

Supplementary File S2. Effects of the length of drying time on the LSVs of lice and fleas stored in alcohol for four years. (A) Proportion of relevant identification of *P. humanus corporis* (green line) and *Ct. felis* (blue line) specimens stored in alcohol for 48 months, according to the length of drying time**. (B)** Comparison of LSVs from *P. humanus corporis* and *Ct. felis* stored in alcohol for 48 months and dried for three different lengths of time. Significant differences in LSVs between duration of drying time are indicated (Kruskal-Wallis test). The dashed line represents the threshold value for reliable identification (LSV > 1.8). a.u., arbitrary units; LSV, log score value.
