# Supplementary material for: Evolution of MALDI-TOF MS Profiles from Lice and Fleas Preserved in Alcohol over Time
Source: Insects. 2023 Oct 20;14(10):825. doi: 10.3390/insects14100825 (PMC10607003; doi:10.3390/insects14100825)
Supplement: Supplementary file 1 [file insects-14-00825-s001.zip › Supplementary Table S1.pdf]

Supplementary Table S1. List of the arthropod species present in our homemade MALDI-TOF MS database.

Arthropod and mode of preservation	Body part	Species present in our reference database
**Fresh or frozen ticks**	Legs	*Amblyomma variegatum*, *Rhipicephalus sanguineus* s.l, *Rhipicephalus bursa*, *Rhipicephalus pulchellus*, *Rhipicephalus sulcatus*, *Hyalomma marginatum rufipes*, *Hyalomma marginatum marginatum*, *Hyalomma impeltatum*, *Hyalomma aegyptium*, *Ixodes ricinus*, *Ixodes persulcatus*, *Dermacentor marginatus*, *Dermacentor reticulatus*, *Dermacentor silvarum*, *Haemaphysalis leachi*, *Haemaphysalis concinna*, *Haemaphysalis japanica*, *Haemaphysalis parva*, *Haemaphysalis punctata*, *Argas persicus*, *Argas lagenoplatis* and *Ornithodoros savignyi*
**Ticks stored in alcohol**	Legs	*Am. variegatum*, *Amblyomma gemma*, *Amblyomma cohaerens*, *Amblyomma compressum*, *Amblyomma exornatum*, *A. persicus*, *Haemaphysalis inermis*, *Hae. leachi*, *Hae. paraleachi*, *Hae muhsamae*, *Hyalomma anatolicum anatolicum*, *Hyalomma detricum*, *Hyalomma dromedarii*, *Hyalomma excavatum*, *Hy. truncatum*, *Hy. m. rufipes*, *Hyalomma aegyptium*, *Ixodes cumulatimpunctatus*, *Ixodes hexagonus*, *I. ricinus*, *Ornithodoros sonrai*, *Rhipicephalus annulatus*, *Rhipicephalus Boophilus decoloratus*, *Rhipicephalus Boophilus microplus*, *Rhipicephalus bergeoni*, *Rh. bursa*, *Rhipicephalus complanatus*, *Rhipicephalus congolensis*, *Rhipicephalus* evertsi *evertsi*, *Rhipicephalus lunulatus*, *Rhipicephalus praetextatus*, *Rh. pulchellus*, *Rhipicephalus pusillus* and *Rh. sanguineus* s.l
**Fresh or frozen ticks infected by bacteria**	Legs	*D. marginatus* infected with *Rickettsia slovaca*, *Rh. sanguineus* infected with *Rickettsia conorii and Rickettsia massiliae*
**Fresh or frozen adult mosquitoes**	Legs	*Aedes aegypti*, *Aedes albopictus*, *Aedes altermans*, *Aedes australis*, *Aedes caspuis*, *Aedes cinereus*, *Aedes difouri*, *Aedes flavifrons*, *Aedes flowri*, *Aedes multiplex*, *Aedes polynesiensis*, *Aedes vexans*, *Aedes vigilax*, *Aedes excrucians*, *Anopheles arabiensis*, *Anopheles coustani*, *Anopheles claviger*, *Anopheles coluzzii*, *Anopheles funestus*, *Anopheles gambiae*, *Anopheles hyrcanus*, *Anopheles maculipennis*, *Anopheles pharoensis*, *Anopheles rufipes*, *Anopheles wellcomei*, *Anopheles ziemanni*, *Culex annulirostris*, *Culex australicus*, *Culex insigni*, *Culex modestus*, *Culex neavei*, *Culex orbostiensis*, *Culex pipiens*, *Culex quinquefasciatus*, *Culex rima*, *Culex sitiens*, *Culex watti*, *Culiseta longiareolata*, *Lutzia tigripes*, *Mansonia africana*, *Mansonia uniformis*, *Ochlerotatus excrusians*, *Ochlerotatus rusticus*, *Orthopodomyia reunionensis and Verralina funereal*
**Fresh or frozen mosquitoes at aquatic stages**	Whole	*Ae. Aegypti*, *Ae. Albopictus*, *An. Gambiae*, *culex hortensis*, *Cx. Modestus*, *Cx. Pipiens*, *Cu. Longiareolata* and *Ochlerotatus caspuis*
**Fresh or frozen lice**	Cephalo-thorax	*Damalinia bovis*, *Damalinia caprae*, *Damalinia ovis*, *Goniodes gigas*, *Goniocotes gallinae*, *Gonoides meleagridis*, *Haematopinus eurysternus*, *Linognatus africanus*, *Linognatus vituli*, *Lipeurus caponis*, *Menacanthus stramineus*, *Menopon gallinae*, *Pediculus humanus corporis* and *Solenopotes capillatus*

**Lice stored in alcohol**	Cephalo-thorax	*Bovicola caprae*, *G. gallinae*, *G. gigas*, *Goniodes dissimilis*, *H. eurysternus*, *H. suis*, *Menacanthus stramineus*, *L. vituli*, *M. gallinae* and *P. humanus corporis*
**Fresh or frozen fleas**	Cephalo-thorax	*Xenopsylla cheopis* and *Ctenocephalides felis*
**Fleas stored in alcohol**	Cephalo-thorax	*Archaeopsylla erinacei*, *C. felis*, *Ctenocephalides canis*, *X. cheopis*, *Leptopsylla taschenbergi*, *Nosopsyllus fasciatus*, *Pulex irritans* and *Stenoponia tripectinata*
**Fresh or frozen bedbugs**	Head	*Cimex lectularius, Cimex hemipterus and Cimex (oeciacus) hirundinis*
**Dry Triatominae**	Legs	*Eratyrus mucronatus*, *Panstrongylus geniculatus*, *Rhodnius prolixus*, *Rhodnius pictipes*, *Rhodnius robustus* and *Triatoma, infestan*
**Fresh or frozen Triatominae**	Legs	*Rhodnius robustus* and *Triatoma infestan*
**Fresh or frozen Sand flies**	Cephalo-thorax	*Phlebotomus papatasi*, *Phlebotomus longicuspis*, *Phlebotomus perfiliewi*, *Phlebotomus perniciosus*, *Phlebotomus sergenti* and *Sergentomyia minuta*
**Mites**	Cephalo-thorax	*Leptotrombidium chiangraiensis*, *Leptotrombidium imphalum*, *Leptotrombidium deliense* and *Dermanyssus gallinae*
**Termitidea**	Legs	*Amitermes evuncifer, Ancistrotermes cavithorax, Cubitermes orthognatus, Kallotermes flavicollis, Macrotermes belicosus, Macrotermes herus, Macrotermes ivorensis, Macrotermes subyalinus, Microcerotermes parvu, Odontotermes latericuis, Procubitermes sjostdie, Promirotermes holmegrinie, Reticulitermes lucifugus, Trinervitermes geminatus, Trinervitermes occidentalis and Trinevitermes trinervis*
**Fresh or frozen Blattidae**	Legs	*Supella longipalpa, Periplaneta americana, Blatta orientalis, Blatella germanica, and Blaptica dubia,*
**Mosquito blood meals**	Abdomen of engorged mosquitoes	*An. gambiae Giles fed on Homo sapiens, Equus caballus, Ovis aries, rabbit, Balb/C. mouse, Rattus norvegicus, Canis, familiaris, Bos taurus, Capra hircus, Gallus gallus, Equus asinus, Tapirus indicus, Tapirus terrestris, Carollia perspicillata, Thraupis episcopus, Erythrocebus patas and Callithrix pygmaea blood, Ae. albopictus fed on Homo sapiens blood*
